# Solid organ transplantation: solid but not yet spectacular

**DOI:** 10.1172/JCI176856

**Published:** 2024-01-02

**Authors:** Laurence A. Turka

**Affiliations:** Harvard Medical School, Massachusetts General Hospital, Boston, Massachusetts, USA.

As a former editor-in-chief of this journal, it is a particular pleasure to provide a contribution in recognition of the *JCI*’s 100th anniversary. My invitation asked me to provide a perspective on “solid organ transplantation/immunosuppression.” In a nutshell, most of what I have to say on the subject is actually captured in that phrasing. Let me explain.

## Early work in solid organ transplantation

Solid organ transplantation was surgically possible before it could be medically successful. The pioneering surgeon Alexis Carrel won the Nobel Prize in 1912 for the development of vascular anastomosis in 1902 (interestingly, he later co-invented organ perfusion pumps with the aviator Charles Lindbergh). However, the first organ allografts (transplants between genetically different individuals, as opposed to isografts, transplants between identical twins) to be modestly successful were not performed until half a century later, the late 1950s, and were enabled by the development of chemical immunosuppression to prevent rejection. An early report of what occurred in a series of nine cases of renal transplantation without immunosuppression was published in the *JCI* (1). Most of the kidneys never functioned, although a few lasted for up to 5 weeks. It’s remarkable to read that, “It was our aim in this investigation to study the subject of homotransplantation (the term then in use) – *not to offer a therapeutic procedure* (emphasis mine).” It’s difficult to imagine how informed consent was obtained.

What changed everything was the development of relatively effective and safe immunosuppression. I used to tell medical students, residents, and fellows that we could completely prevent rejection in every patient but that they would die of overwhelming infection very quickly. The discovery of purine antagonists (originally developed for chemotherapy, another Nobel Prize–winning achievement) ushered in a two-decade era from the early 1960s to the early 1980s when azathioprine and corticosteroids were used for prophylaxis of rejection. One-year renal allograft survival was 40%–50%. This outcome is certainly unacceptable by today’s standards, but it was quite remarkable at the time. Other organs were not yet routinely transplanted. Another giant step forward came with the discovery of the first calcineurin inhibitor, cyclosporine, by scientists at Sandoz (now Novartis) (2) and its application to clinical practice, led in renal transplantation by Roy Calne and in liver transplantation by Tom Starzl. Renal allograft survival increased with a quantum leap, topping 80% (and now exceeding 90% in previously untransplanted patients), and transplantation of other organs, such as liver and heart, became much more practical.

## Progress made but substantial barriers remain

The problem that I started studying when I first began my career as a physician-scientist remains unsolved to this day — inducing tolerance to solid organ transplants so as to obviate the need for ongoing nonspecific immunosuppression. While immunosuppressive regimens, and graft survival, have continued to improve throughout my career in medicine, most grafts are eventually lost to some form of rejection. Moreover, to varying degrees, patients are burdened with the substantial side effects of the drugs they take to prevent rejection. These include nephrotoxicity, cardiovascular disease, diabetes, osteoporosis/necrosis, and opportunistic infection. While some of these are off-target effects, susceptibility to infection is not. It is intrinsically impossible to divorce opportunistic infection from effective immunosuppression, because immunosuppression is designed to suppress your immune system!

If achievable, antigen-specific tolerance to a transplant would obviate the need for immunosuppression and likely also achieve much longer graft survival. Can it be done? In theory, it would seem so. Let’s rewind to the 20th century again. In 1945 Ray Owen, then a newly minted assistant professor at the University of Wisconsin, made the truly remarkable observation that when two genetically different calves were found to share the same placenta in utero, so-called Freemartin cattle, they did not reject skin transplants from each other when transplanted as adults (3) (as a side note, the skin is the most stringent test of transplantation tolerance). This tolerance was due to naturally acquired chimerism, as each calf was exposed to blood cells from its sibling in utero. Many (including this author) believe that Owen did not get the recognition he deserved for this finding, but it did not escape the attention of Peter Medawar, who hypothesized that when the immune system was developing, it could acquire tolerance to the antigens that it “saw.” His seminal paper, “Actively acquired tolerance of foreign cells” published in *Nature* in 1953 (4), demonstrated that one could actively exploit this capability to create acquired immune tolerance; it earned Medawar, but not Owen, a share of a Nobel Prize, along with Macfarlane Burnet, who predicted that acquired immune tolerance could occur via this mechanism. Since then, tolerance has been the ultimate, yet elusive, goal in transplantation.

What makes transplantation tolerance so difficult to achieve? Here I point to a number of factors.

### The response to alloantigens.

For reasons that have been well reviewed in the past, the response to alloantigens is quantitatively strong. Roughly 1%–5% of T cells are alloreactive, compared with roughly 0.001% that are reactive to any given peptide antigen (5).

### Memory T cell responses are particularly robust.

This fact should not be surprising, and it is part of why vaccines work to modulate disease even if infection is not prevented. Even in patients who have never been transplanted before, many of the alloreactive cells will be memory cells because of cross-reactivity with other antigens to which the patient has been exposed (e.g., viral antigens). Memory T cells increase with age, as does organ failure and the need for transplants (6).

### Lack of suitable animal models.

The immune system in rodents is much easier to manipulate than that in humans. While the results of studies in nonhuman primates have a much higher predictive value, ethical and financial constraints preclude their routine use. Many interventions have been shown to induce tolerance in mice and rats. To my knowledge, only one has translated into humans. This approach, the induction of mixed hematopoietic chimerism via bone marrow or allogeneic stem cell transplantation, has seen notable success in a small number of patients. But it has not been widely studied or adapted, in part because of the complex nature of the regimen and associated toxicity (7).

### The relative success of immunosuppression.

Current immunosuppression is characterized by outstanding short-term results (a credit to years of work by biomedical researchers in academia and industry) with minimal toxicity during that period. This outcome makes patients and physicians understandably reluctant to participate in tolerance trials because the risk/benefit ratio can be too high. An exception is liver transplantation. The liver is both naturally relatively tolerogenic and also capable of repair from injury in ways that the kidneys, heart, and lungs are not. Most tolerance trials are therefore performed in this clinical setting.

### A lack of good biomarkers.

The only reasonable way to balance patient safety in tolerance studies is to utilize immunosuppression at the outset and gradually withdraw it. One way to facilitate tolerance trials would be to apply biomarkers that identify which patients can be safely weaned from their medications. Studies to identify such biomarkers are challenging, as tolerant patients are rare. A number of possible candidate markers have been identified (8, 9), but unfortunately confirmatory studies have not been performed. An overwhelming number of things *can* be measured. Broad profiling approaches and novel analytic methods like AI may be helpful in sorting the wheat from the chaff.

## What does this mean for the future?

Our current understanding is that a combination of deletion of antigen-reactive cells, as well as active immunoregulation of the remaining cells, is the mechanism that creates and maintains immune tolerance. Because the frequency of alloreactive cells is so high (compared with, for example, that of autoreactive cells), it seems likely that tolerance may require a strong element of deletion as well as continued regulation to control nondeleted cells, plus new T cells that emerge from the thymus (10, 11). Most likely, deletion can/will occur during the “induction” phase of therapy, i.e., peritransplant. During this time many alloreactive T cells become activated and susceptible to targeting. Specific deletion of alloreactive cells, rather than global T cell deletion, may be important to prevent lymphopenia-induced expansion of residual cells, which induces memory differentiation (12). Ways to promote immunoregulation (or exhaustion of alloreactive cells) will be needed in the “maintenance” phase of treatment. Low-dose IL-2 or IL-2 mimetics designed to activate Tregs but not effector cells may be one such approach. A variety of efforts are also underway using adoptive cell therapy with autologous Tregs, which can be polyclonal, enriched for antigen-specific cells in culture (13), or made antigen specific via the use of a CAR (14) or a transgenic T cell receptor. We can also anticipate trials in cellular transplantation using genetically engineered porcine islets or induced pluripotent stem sells differentiated into islets or hepatocytes and simultaneously modified to make them relatively “immunosilent.”

Compared with where the field was 100 years ago, progress has been truly spectacular. Compared with where we would like to be, it’s been... solid, but, honestly, there is nothing wrong with that.

## Version 1 01/02/2024

Electronic publication
